# Biotechnological Prospects of *Thermoanerobacter* AK15: End-Product Formation from Carbohydrates, Amino Acids, and Lignocellulosic and Macroalgae Hydrolysates

**DOI:** 10.3390/ijms25063490

**Published:** 2024-03-20

**Authors:** Johann Orlygsson, Sean Michael Scully

**Affiliations:** Faculty of Natural Resource Sciences, University of Akureyri, Borgir, Nordurslod 2, 600 Akureyri, Iceland; scully@unak.is

**Keywords:** *Thermoanaerobacter* carbohydrate, protein, sugar, amino acid, fermentation products

## Abstract

The conversion of lignocellulosic and algal biomass by thermophilic bacteria has been an area of active investigation. *Thermoanaerobacter* species have proven to be particularly capable in the production of bioethanol and biohydrogen from lignocellulosic biomass, although detailed studies of their abilities to utilize the full gamut of carbohydrate, amino acids, and proteins encountered in biomass hydrolysates are seldom comprehensively examined. Here, we re-evaluate the ability of *Thermoanaerobacter* strain AK15, a highly ethanologenic strain previously isolated from a hot spring in Iceland. Similar to other *Thermoanaerobacter* species, the strain degraded a wide range of mono- and di-saccharides and produced a maximum of 1.57 mol ethanol per mol of glucose degraded at high liquid–gas phase ratios. The ability of strain AK15 to utilize amino acids in the presence of thiosulfate is limited to the branched-chain amino acids as well as serine and threonine. Similar to other *Thermoanaerobacter* species, strain AK15 produces a mixture of branched-chain fatty acids and alcohols, making the strain of interest as a potential source of longer-chain alcohols. Finally, the strain was also shown to use butyrate as an electron sink during glucose degradation resulting in the reduced product butanol, in addition to end-products produced from glucose. Thus, strain AK15 is a promising candidate for ethanol and higher-order alcohols from a range of lignocellulosic and algal biomass.

## 1. Introduction

Production of both biofuels and fine chemicals from renewable sources is in more demand than ever before. The urge to replace fossil fuels with renewable alternatives is becoming more and more urgent because of the global climate changes on Earth as well as the geopolitical uncertainties associated with major oil exporting nations. As such, the United Nations Sustainable Development Goals place a great emphasis on climate action and sustainable development.

While there are several viable options for biofuels that can meet the needs of a liquid energy carrier to replace gasoline, bioethanol and other alcohols are promising due to their ease of production, low toxicity, direct compatibility of blended fuels with many existing combustion engines, and high volatility [1]. Furthermore, bioethanol production has enjoyed a long history, but its production as a biofuel at industrial scales has been primarily limited to using biomass containing starch and sucrose as a substrate, which poses several problems, namely the environmental impact and negligible energy yield from cultivating these crops and increasing the price of food [2,3]. To this end, there has been a lot of interest in using other biomasses that do not compete with food utilization, such as lignocellulosic and seaweed biomass. Lignocellulosic biomass is composed of cellulose, hemicellulose, and lignin [4], while seaweeds have a much more diverse composition of carbohydrates, which often includes cellulose but can also include β-glucans, such as laminarin and uronic-acid containing carbohydrates (e.g., carrageenan, alginate, agarose, mannitol, and sulfated polysaccharides) [5,6]. As with terrestrial biomass, the exact composition varies as a function of the type of seaweed, in addition to other variables related to growth conditions. Both lignocellulosic and algal biomass present a challenge to the production of bioethanol, given the greater diversity of carbohydrate building blocks present, making finding an organism for bioprocessing more challenging.

The production of bioethanol from simple biomass has also led to the debate on the utilization of food and feed for fuel production. Therefore, the utilization of more complex biomass is a better alternative, but its use is more complex because of the complex structure and the pretreatment needed for their degradation to hexoses and pentoses. Additionally, the most common microorganism used for bioethanol production has a limited capacity for degrading the wide variety of sugars present in lignocellulosic biomass. This has led to a search for more feasible microorganisms with a broader substrate range than yeasts. To this end, thermophilic anaerobes have been promising candidates for this purpose, and many studies in the past twenty years have focused on hydrogen and ethanol production from complex biomass [2,3,7,8]. Additionally, several species belonging to the genera *Thermococcus*, *Pyrococcus*, *Caldicellulosiruptor*, and *Thermotoga* have been genetically manipulated to increase hydrogen and/or ethanol production [9,10,11,12]. Production of fine chemicals, like 1,2-propanediol, 1,3-propanediol, and branched-chain alcohols, have historically been produced from non-renewable sources, like gasoline and gases [13]. More environmental routes to such chemicals are thus more feasible and have led to the investigation of the production of various fine chemicals [14]. Several thermophilic bacteria have been used for the production of various fine chemicals, e.g., *Thermoanaerobacterium* and *Caldicellulosiruptor* for 1,2 propanediol [15,16], *Caloramator* for 1,3 propanediol [17], and *Thermoanaerobacter* and *Caldanaerobacter* for the production of branched-chain alcohols [18]. Thermophiles have also been described as emerging metabolic platform organisms for the production of various industrial products apart from ethanol and hydrogen, e.g., for phytoene, acetoin, 3-hydroxypropionate, and butanol [19].

All species within *Thermoanaerobacter* are obligate anaerobes—they ferment various carbohydrates to ethanol, acetate, lactate, hydrogen, and carbon dioxide [20] and originate from various habitats like hot springs, hydrothermal vents, and oil fields [21,22,23,24]. Species within the genus *Thermoanaerobacter* have received considerable attention due to their biotechnological potential, having a very broad substrate spectrum, especially among the sugars present in lignocellulosic biomass, and high ethanol yields from lignocellulosic biomasses [25,26]. Many *Thermoanaerobacter* species have been investigated for their ethanol production, e.g., *T. ethanolicus*, *T. mathranii,* and *T. pseudethanolicus* [7,8,27]. Recent investigations have also shown the ability of some species within the genus to produce valuable high-carbon alcohols from specific amino acids [18], as well as to reduce fatty acids to their corresponding alcohol during growth on both sugars and amino acids [28]. It is of interest that the end-products formed during fermentation can be manipulated by controlling environmental factors. Thus, a very good ethanol-producing strain can be cultivated under conditions that may lead to acetate and hydrogen as the main end-products. This has been shown for *Thermoanaerobacter* strain AK5, where the ethanol production varied from 0.7 mol ethanol/mol glucose to 1.7 mol ethanol/mol glucose simply by cultivating the strain at different liquid to gas phase ratios [29]. Similarly, the acetate produced changed from 0.14 to 0.74 mol acetate/mol glucose. This change was even more dramatic when the strain was cultivated in the presence of thiosulfate or in the presence of a hydrogenotrophic methanogen. Under these conditions, ethanol became only a minor end-product but acetate, by far, was the major volatile end-product. Due to the broad substrate range of *Thermoanaerobacter*, they have often been linked to biofuel production from complex biomass (lignocellulose). Several reports show that *Thermoanaerobacter* species produce between 3 and 6 mM ethanol g^−1^ dw of various biomass types, such as grass, hemp, barley straw, corn straw, and so forth ([3] and references therein).

The utility of thermophilic anaerobes as bioprocessing organisms has been well established, with *Thermoanaerobacter* strains being particularly useful for bioethanol and other alcohol production from complex biomass, although there are substantial gaps within the utilization of a wide range of carbohydrates and the utilization of amino acids is often completely overlooked. The present investigation aims to provide a comprehensive examination of *Thermoanaerobacter* strain AK15’s ability to utilize the carbohydrates present in a wide range of lignocellulosic and algal biomass, as well as the strain–s ability to utilize proteogenic amino acids and proteins. Additionally, *Thermoanaerobacter* strain AK15–s potential to utilize complex biomass and macroalgae hydrolysates is explored. *Thermoanaerobacter* strain AK15 is a strictly anaerobic bacterium and was isolated from an alkaline hot spring (60 °C, pH 8.6) in Viti in the Krafla area in NE Iceland, as previously described by Scully and co-workers [18].

## 2. Results and Discussion

### 2.1. Substrate Spectra

*Thermoanaerobacter* AK15 was cultivated on the main carbohydrates present in lignocellulosic and algal biomass, including hexoses (glucose, mannose, galactose), pentoses (arabinose, xylose), disaccharides (cellobiose), as well as several deoxysugars (L-fucose and L-rhamnose), and mannitol, which is found in seaweeds. Additionally, strain AK15 was tested for growth on polymeric carbohydrate substances, all 20 of the proteogenic amino acids with and without the inclusion of thiosulfate (20 mM), and various proteins with and without thiosulfate (20 mM).

A summary of the selected features of *Thermoanaerobacter* strain AK15 compared to selected strains within the genus is presented in Table 1.

#### 2.1.1. Degradation of Carbohydrates

**Figure 1:**
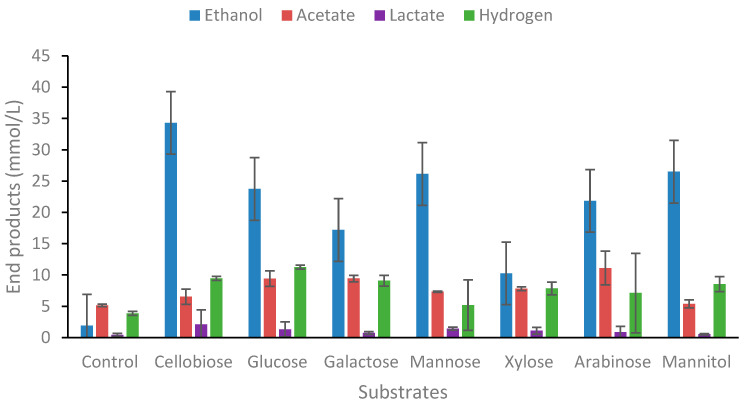
End-product formation from various substrates. From left to right, acetate, ethanol, lactate, and hydrogen. Values are the average of three replicates with standard deviations presented as error bars.

Members of the genus often degrade most of the hexoses and pentoses common to lignocellulose, although the ability to utilize arabinose and mannitol is somewhat variable between species. Here, the ability of strain AK15 to utilize hexoses, pentoses, deoxyhexoses, sugar alcohols, and disaccharides was investigated in batch culture. *Thermoanaerobacter* AK15 degraded cellobiose and all tested monosaccharides except for the two deoxysugars, L-fucose and L-rhamnose (Figure 1). The main end-product in all substrates that were degraded was ethanol (Figure 1), which resulted in optical densities between 0.35 and 0.48 and a decrease in pH of about 1.0 pH unit compared with the control (Supplementary Table S1). Some thermophiles have been shown to degrade deoxysugars, like *Caldicellulosiruptor* species, of which more than half of the type strains within the genus degraded L-rhamnose to 1,2-propanediol and three strains degraded L-fucose to 1,2-propanediol [16]. Other thermophilic anaerobes known to produce 1,2-propanediol are moderately thermophilic strains of *Clostridium* [33,34], although, in this instance, 1,2-propanediol was not a detected end-product.

Similar to other *Thermoanaerobacter* strains, hexoses and pentoses were fermented to ethanol as the dominant end-product. Arabinose, which is less commonly used as a substrate, was fermented, although xylose was poorly utilized. Notably, mannitol utilization was not tested in the original characterization study of Wagner and co-workers describing *T. uzonensis* [32], although, in the present work, the strain degraded mannitol to ethanol as the dominant end-product, as has also been demonstrated for *T. uzonensis*, the closest relative to strain AK15 [31].

The original data on ethanol production from glucose for *T. uzonensis* showed 1.15 mol ethanol per mol glucose degraded [32], while *Thermoanaerobacter* strain AK15 produces 1.10 ethanol mol glucose^−1^ (Figure 1). Other well-known species producing high amounts of ethanol from carbohydrates within the genus are *T. ethanolicus* and *T. pseudethanolicus* [8,27]. The other end-products are mainly acetate with traces of lactate; this was also the case for other carbohydrates tested. Of particular interest is the strain–s ability to degrade mannitol. While three pathways for the catabolism of mannitol have been described to date [35], it is most likely that strain AK15 uses mannitol via phosphorylation using a PEP-dependent phosphotransferase and subsequent reduction to fructose-6-phosphatase via mannitol-1-phosphate dehydrogenase, as has been reported for other solventogenic clostridia [36].

**Figure 2:**
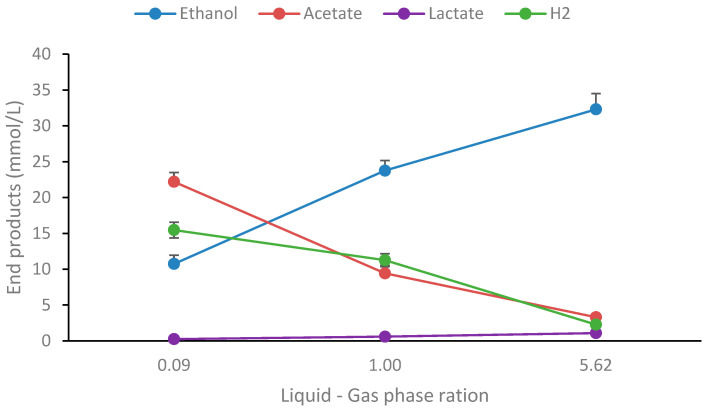
End-product formation from glucose (20 mM) at different liquid–gas phase ratios. Values are the average of three replicates with standard deviations presented as error bars.

It is a well-known phenomenon that the ratio of end-products is highly influenced by culture conditions, particularly hydrogen accumulation. Therefore, a simple experiment was performed on the strain by cultivating it in three different liquid–gas phase ratios: 0.09, 1.00, and 5.62. As shown in Figure 2, the end-product profile changes dramatically during growth under different liquid–gas phase ratios for the strain.

At high *p*H_2_, the main end-product is ethanol, or 33.3 mM, which corresponds to 1.57 mol/mol glucose degraded (controls subtracted), but it drops to 0.44 mM at the lowest *p*H_2_. Similarly, acetate and hydrogen decrease at high *p*H_2_ conditions. The higher acetate concentrations at low liquid–gas phase ratios resulted in lower pH, but the optical density was similar in all conditions (Supplementary Table S2). This highlights the importance of the culture conditions on end-product formation and has been reported for various thermophilic bacteria in recent studies [29,37]. Thus, the strain is highly ethanologenic and can be manipulated to convert more than 75% of the glucose to ethanol, as has been reported for several other *Thermoanaerobacter* strains [38]. Also, being versatile concerning the utilization of a wide variety of carbohydrates, strain AK15 may be considered a good candidate for bioethanol production from lignocellulosic biomass. To investigate this further, the strains were cultivated on several lignocellulosic and algal biomasses pretreated with dilute acids and cellulases.

#### 2.1.2. Degradation of Polymeric Carbohydrate Substrates

**Figure 3:**
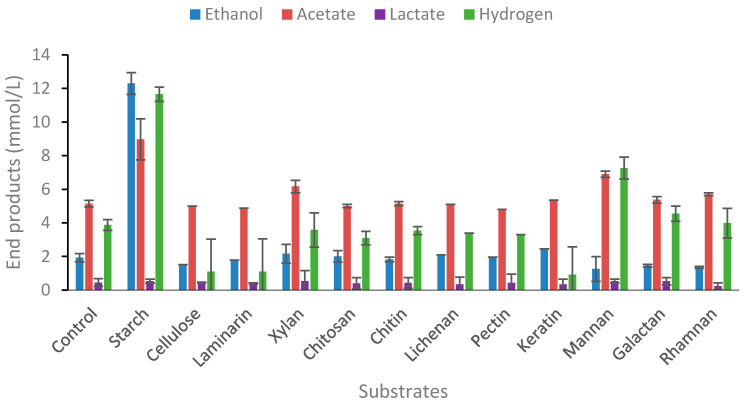
End-product formation from various polymeric substrates. Values are the average of three replicates with standard deviations presented as error bars.

The ability of microorganisms to be good candidates for bioprocessing of lignocellulosic or algal biomass should include the capability of degrading a wide range of glycosidic bonds. Several, but not all, *Thermoanaerobacter* strains can degrade starch, pectin, and xylan, although the ability to degrade lichenan and other beta-glucans has been seldom reported. The ability of strain AK15 to degrade β-1,3-*O* glycosidic bonds was evaluated using lichenan and laminarin. The ability of strains to utilize the hemicellulose fraction was evaluated using xylan, mannan (β-*O*-(1-4) linked mannose), galactan, and rhamnan. The strain did not utilize any of these substrates. Of the polymeric carbohydrates tested, strain AK15 only showed end-product formation from starch (Figure 3). *T. uzonensis* is, however, reported as starch-negative [32], although many of the members of the genus degrade starch as the sole carbon source. The main end-product from starch was ethanol (12.3 mM). No growth was observed when the strain was cultivated on cellulose, as previously reported for *Thermoanaerobacter* species [20], nor did the strain degrade laminarin, chitosan, lichenan, keratin, mannan, galactan, or rhamnan. This is also confirmed by no increase in optical densities or lowering of pH in the cultures (Supplementary Table S3). There are only several species within the genus that are known to degrade keratin, with the best-known being “*Thermoanaerobacter keratinophilus*” [39], although this strain likely falls within the modern genus of *Caldanaerobacter*.

#### 2.1.3. Degradation of Biomass Hydrolysates

**Figure 4:**
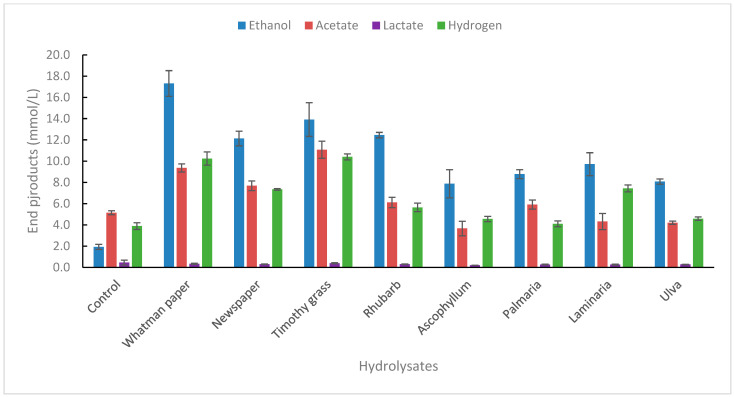
End-product formation from hydrolysates made from lignocellulosic and macroalgae biomasses. The concentration of hydrolysates was 2.5% (*v*/*v*). Values are the average of three replicates with standard deviations presented as error bars.

The strain was tested on several complex biomass types, both lignocellulosic biomass (grass, newspaper, rhubarb leaves) and macroalgae types (*Ascophyllum nodusum*, *Palmaria palmata*, *Laminaria digitata*, *Ulva lactuca*), as shown in Figure 4. Whatman paper was used as a reference in the same concentration as for the biomass samples.

As for the end-product formation from individual sugars, the main end-product from the biomass hydrolysates used was ethanol. The strain was positive for end-product formation (Figure 4) and growth (Supplementary Table S4). Glucose degradation under the same conditions resulted in the formation of 23.8 mM of ethanol, or 1.15 mol/mol glucose degraded. Theoretical yields of glucose from 2.5% *w*/*v* Whatman paper that is completely hydrolyzed is 15.4 mM. The amount of ethanol produced from Whatman paper hydrolysates is 15.4 mM, or 1.0 mol ethanol/mol glucose, which corresponds to 5.6 mM/g glucose, assuming all glucose is released during hydrolysis (control ethanol production subtracted). Not surprisingly, ethanol yields were lower in lignocellulosic biomasses (newspaper, grass, rhubarb) because of its more complex nature compared with mono- and disaccharides [40]. The yields on newspaper, grass, and rhubarb are 3.7, 4.4, and 3.9 mM g^−1^ dw. The highest yields of ethanol from complex biomass by thermophilic bacteria is by *Thermoanaerobacter* strain BG1L1 on wheat straw and corn stover, or 8.5–9.2 mM ethanol g^−1^ dw [8,41]. Yields from grass are relatively good compared with other similar species. *Thermoanaerobacter* strain AK5 produces 4.1 mM g^−1^ dw grass [29], and *Thermoanaerobacterium* strain AK17 produces 5.5 mM g^−1^ dw [42]. In all cases, the grass hydrolysates were pretreated with acid, as well as with enzymatic pretreatment, as performed in the present study. Many other reports on thermophiles capable of good ethanol yields from lignocellulose have also been presented in recent years [30,43,44,45,46]. Thus, *Thermoanaerobacter* strain AK15 seems to be suitable for bioethanol production from complex biomass with good ethanol yields.

Macroalgae is another type of biomass that may be of future use as a potential biomass for bioethanol and bioactive compound production [47,48,49,50]. In the present study, four types of macroalgae were used, two brown algae (*Ascophyllum nodusum*, *Laminaria digitata*), one red algae (*Palmaria palmata*), and one green algae (*Ulva lactuca*). Ethanol yields for these macroalgae were, in most cases, lower compared with lignocellulosic biomass. The highest yields were from *Laminaria digitata*, or 2.8 mM g^−1^ dw, which corresponds to 25% of theoretical yields. Most data on ethanol production from various macroalgae species in the literature originate from yeast fermentation of hydrolysates produced by a wide variety of pretreatment methods. Hou and co-workers reported the production of 14.7 gL^−1^ of ethanol using an SSF approach involving cellulase and alginate lyase of *L. digitata* (10 g/L) using *S. cerevisiae* as a fermenting microorganism [51]. Similarly, Lee and co-workers achieved an ethanol titer of 6.65 gL^−1^ of ethanol from a cellulase hydrolysate of *S. japonica* (60 g/L) using *S. cerevisiae* strain DK 410362 [52], while others achieved a 14.0 gL^−1^ ethanol titer from *S. japonica* waste [53]. A report by Wang et al. used *Gracilaria salicornia*, an invasive brown algae, to achieve a modest ethanol titer of 1.57 g/L using *E. coli* strain KO11 (ATCC 55124) [54]. A recent investigation of the fermentation of *Laminaria digitata* hydrolysates, pretreated with enzymatic hydrolysis, showed 0.30 gg^−1^ of consumed substrate (59% of theoretical yields) [55].

#### 2.1.4. Degradation of Amino Acids

The is a large amount of data concerning the sugar metabolism of thermophilic bacteria in general, with most focusing on ethanol production from complex biomass. Much less attention has been focused on the capacity of thermophiles to degrade amino acids. Most of the information on amino acid catabolism among Clostridia has been gained from well-known proteolytic members of *Clostridium sporogenes* [56], *Clostridium botulinum* [57], and *Clostridium sticklandii* [58]. The degradation of amino acids is a complex process involving several oxidation and reduction steps, and only possible under specific conditions; unlike carbohydrates, many amino acids are not degraded as single substrates because of the thermodynamics involved in the reactions [59]. There are various pathways to degrade the 20 amino acids present in proteins, some of which are highly reduced and are not degraded unless the electrons produced in the oxidative step are scavenged [60,61,62]. The most common way to degrade amino acids is to use a two-step mechanism. First, an oxidative deamination of the amino acid yields a corresponding keto acid, which is oxidatively decarboxylated to give one carbon shorter fatty acids [59]. This is possible under anaerobic conditions only for amino acids with high oxidation stages [59]. The so-called reduced amino acids, e.g., the branched-chain amino acids (leucine, isoleucine, valine) and alanine. In the 1990s, several investigations showed that the reduced amino acids could only be degraded when the amino acid-degrading bacteria could dispose of the electrons produced during the oxidation of these amino acids to an external electron acceptor. This could be done either by co-cultivating the amino acid degrading bacterium with a hydrogenotrophic bacterium, i.e., methanogens, via interspecies hydrogen transfer, or by using thiosulfate as a chemical electron acceptor. This was shown by *Thermoanaerobacter brockii* during growth on the branched-chain amino acids [63], where leucine, isoleucine, and valine were degraded to 3-methylbutyrate, 2-methylbutyrate, and 2-methylpropionate, in the presence of thiosulfate, respectively. Later investigations revealed that the branched-chain amino acids were not only degraded to their corresponding fatty acid but to a mixture of their corresponding fatty acid and alcohol by *Thermoanaerobacter brockii* and *Caldanaerobacter subterraneaus* subsp. *yonseiensis*, when thiosulfate was added into the culture medium [64]. In the present investigation, serine and threonine were the only amino acids that were degraded without the addition of thiosulfate, mainly to ethanol and acetate. In the presence of thiosulfate, these two amino acids were also degraded with a change in the proportion of the end-product formation (see Equations (1) and (2) below). In the case of serine degradation, acetate became the main end-product, and ethanol was a minor product. Similarly, acetate became the main end-product in the presence of thiosulfate during threonine degradation, but ethanol increased as well. Equation (1a,b) show the stoichiometry of serine degradation without and with thiosulfate, and Equation (2a,b) for threonine, respectively.

20 mM Serine → 6.91 mM Ethanol + 11.33 mM Acetate(1a)

20 mM Serine + 20 mM S_2_O_3_ → 1.21 mM Ethanol + 21.41 mM Acetate (1b)

20 mM Threonine → 3.42 mM Ethanol + 6.15 mM Acetate(2a)

20 mM Threonine + 20 mM S_2_O_3_ → 10.68 mM Ethanol + 16.70 mM Acetate (2b)

Similarly, *Thermoanaerobacter brockii* shifts its end-product formation during growth on serine in the presence of an electron acceptor, pushing the end-product towards more oxidized end-products like acetate and less reduced end-products, like ethanol [63].

**Figure 5:**
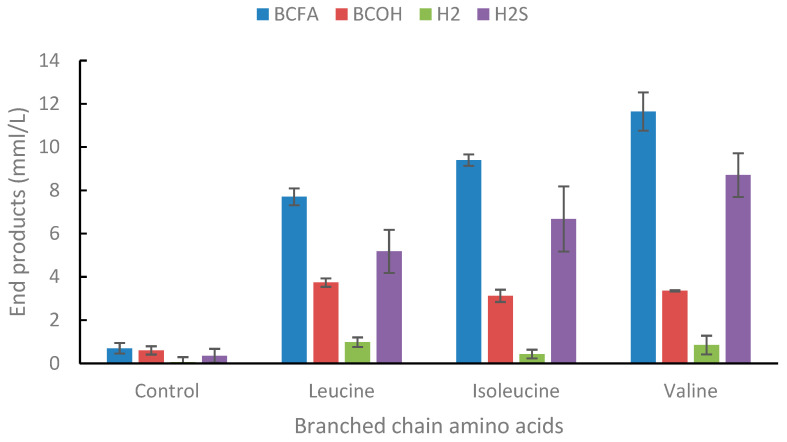
End-product formation from leucine, isoleucine, and valine (all individually at 20 mM concentrations) in the presence of thiosulfate (40 mM). For simplicity, formation of acetate, ethanol, and lactate is not shown but was in similar concentrations as in control bottles. Values are the average of three replicates with standard deviations presented as error bars.

In the present study, the thiosulfate addition resulted in the degradation of the branched-chain amino acids (leucine, isoleucine, and valine) (Figure 5). As previously reported for other *Thermoanaerobacter* strains [18], *Thermoanaerobacter* strain AK15 produced a mixture of branched-chain fatty acids (BCFAs) and branched-chain alcohols (BCOHs) from branched-chain amino acids (BCAAs), and the concentration of the acid was always higher than the alcohol. When the strain was cultivated without the addition of thiosulfate, no growth was observed (Supplementary Table S5).

In the presence of thiosulfate, leucine was degraded to 3-methylbutyrate and 3-methyl-*1-butanol, isoleucine to 2-methylbutyrate and 2-methyl-1-butanol, and valine to 2-methylpropionate and 2-methyl-1-propanol. BCAAs have been reported to be degraded to BCFAs and BCOHs under anaerobic conditions, mostly by *Lactobacillus* and yeasts that use the Ehrlich pathway. Usually, the concentration of these compounds is of importance for the flavor of foods and beverages [65]. Early studies of thermophilic anaerobic bacteria were performed in several investigations on *Thermoanaerobacter brockii*. This bacterium was described to degrade the BCAAs to their corresponding BCFAs, but only when thiosulfate was added to the culture medium, acting as a hydrogen scavenger. The thermodynamics in the deamination of leucine to its corresponding keto acid is ΔG + 51.5 kJ/mol, whereas the ΔG of the amino acid to 2-methybutyrate is +4.2 kJ/mol. Recent work in our laboratory has since shown that, indeed, the main bottleneck for the degradation of these reduced amino acids is the first energy-demanding deamination step. Original investigations also showed that both *Thermoanaerobacter brockii* and *Caldanaerobacter subterraneaus* subsp. *yonseiensis* degraded the branched-chain amino acids to only their corresponding BCFAs when the strains were co-cultivated in the presence of a hydrogenotrophic methanogen but to a mixture of their BCFAs and BCOHs when cultivated alone in the presence of thiosulfate [64]. Thus, the effectiveness of the electron acceptor seems to be important in determining the product distribution during the degradation of BCAAs. Later studies on other strains within the genera of *Thermoanaerobacter* and *Caldanaerobacter* showed that this ability to produce a mixture of alcohols and acids from BCAA was common among both genera [18]. Investigations to understand in more detail the reaction pathway these bacteria use to produce both the acid and the alcohol have been conducted with *Thermoanaerobacter* strain AK85, which is closely related to *Thermoanaerobacter uzonensis*. This study showed that, indeed, the partial pressure of hydrogen was of great importance for the ratio of end-products formed [66]. Finally, it was demonstrated by NMR studies that these bacteria first produce the BCFA, which, in turn, is converted to their corresponding BCOH, both for *Thermoanaerobacter* strain AK85 and *Thermoanaerobacter pseudethanolicus* [66,67]. 

#### 2.1.5. Degradation of Proteins

Protein degradation by thermophilic anaerobic bacteria has received much less attention than carbohydrates. Mesophilic anaerobic protein-degrading bacteria have, however, received much more attention, mainly because of the high number of pathogens that are proteolytic. Studies on thermophilic anaerobes have been limited to several genera, like *Caloramator* and *Thermoanaerobacter*. The importance of electron acceptors for protein and amino acid degradation has been known for some time now and is important knowledge for understanding the role of thermophilic bacteria in hot environments [68].

**Figure 6:**
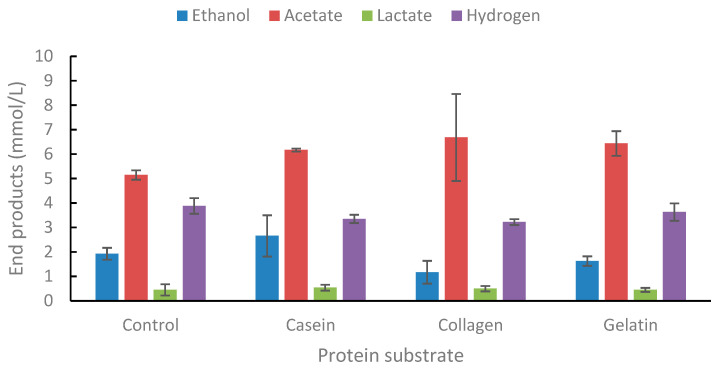
End-product formation from casein, collagen, and gelatin. Concentration is 0.2% (*w*/*v*). Values are the average of three replicates with standard deviations presented as error bars.

The strain was tested for growth on three types of proteins, casein, collagen, and gelatin (Figure 6). A slight increase was observed in acetate from casein and collagen compared to controls. However, the addition of peptone and yeast extract enhanced end-product formation by the strain.

#### 2.1.6. Conversion of Fatty Acids to Alcohols

Recent investigations have shown that bacteria within the genera *Thermoanaerobacter* and *Caldanaerobacter* can dispose of their electrons produced during glucose (and other sugars) oxidation not only to pyruvate to produce ethanol or lactate but may also use other electron acceptors, like fatty acids, which are converted to their corresponding alcohols [28,68,69]. This was tested for strain AK15 by cultivating the strain on glucose only and on glucose in the presence of butyrate. The strain degraded glucose to a mixture of ethanol, acetate, and lactate according to Equation (3):

1 Glucose → 1.24 Ethanol + 0.48 Acetate + 0.05 Lactate + 0.54 H_2_ (3)

When the strain was cultivated on glucose with the addition of 20 mM of butyrate, the reaction stoichiometry changed according to Equation (4).

1 Glucose + 1 Butyrate → 0.78 Ethanol + 0.87 Acetate + 0.58 Butyrate + 0.36 Butanol(4)

Thus, as expected, the strain produces less ethanol and more acetate in the presence of butyrate as an electron acceptor, and the fatty acid is partially converted to its corresponding alcohol, 1-butanol. No lactate and hydrogen were observed as end-products at the end of fermentation in the presence of butyrate. This was also investigated for other volatile fatty acids, like propionate, branched-chain fatty acids, and pentanol, with similar results in the conversion of the fatty acids to their corresponding alcohol. *Thermoanaerobacter pseudethanolicus* has recently been shown to convert fatty acids to alcohols during sugar degradation [69]. The production of high-carbon alcohols from complex biomass by adding cheap volatile fatty acids to the fermentation broth of the hydrolysates is, indeed, a new method of biofuel production that may be of great importance in the near future.

#### 2.1.7. General Discussion

Similar to other *Thermoanaerobacter* strains within the genus, strain AK15 degrades a wide range of carbohydrates and utilizes the branched-chain amino acids and serine and threonine. Although strain AK15 is highly ethanologenic, the titers reported here are relatively low. This can potentially be addressed through the manipulation of culture conditions to maximize the substrate loading and by better exploring the strain’s tolerance to the accumulation of alcoholic end-products, potentially shifting to a fed-batch or continuous fermentation mode as appropriate. Additionally, the deletion of genes that result in the formation of competing reduced end-products, namely hydrogen and lactic acid, may further increase ethanol yields by increasing the pool of reducing potential.

The versatility of strain AK15 makes it a strong candidate for bioprocessing applications from a wide range of lignocellulosic and algal biomass, which often contains a large protein fraction. The bioprocessing of macroalgae, despite its diverse assortment of complex carbohydrates, is a promising alternative feedstock for which strain AK15 may be well suited. As an example, the mannitol found in brown macroalgae is not only abundant but an inexpensive material that could be exploited for bioethanol production or potentially as a source of reducing potential for the reduction of carboxylic acids to alcohols. While the tolerance to initial substrate concentration and alcoholic end-products is yet uninvestigated, this would make a logical next step of investigation, as specific details regarding the mannitol utilization, branched-chain amino acid catabolism, and carboxylic acid reduction require additional investigation, although it is hoped that the whole genome sequence of this organism will offer additional insights.

## 3. Materials and Methods

### 3.1. General Methods

All chemicals were obtained from Sigma-Aldrich (St. Louis, MO, USA) unless otherwise noted. L-fucose and fucoidan were obtained from Dextra (Reading, UK). Starch was from corn. Rhamnogalacturonan (from soy), mannan (from ivory nut), and galactan (from lupin) were obtained from Megazyme (Auchincruive, Scotland). Keratin was locally obtained from chicken feathers, dried and milled, and used without further preparation.

### 3.2. Culture Medium and Preparation

*Thermoanaerobacter* strain AK15 was cultivated in Basal Mineral (BM) medium prepared as previously described [70]; the medium consisted of (per liter): NaH_2_PO_4_ 2.34 g, Na_2_HPO_4_ 3.33 g, NH_4_Cl 2.2 g, NaCl 3.0 g, CaCl_2_ 8.8 g, MgCl_2_ × 6 H_2_O 0.8 g, yeast extract 2.0 g, resazurin 1 mg, trace element solution 1 mL, vitamin solution (DSM141) 1 mL, and NaHCO_3_ 0.8 g. The trace element solution consisted of the following on a per liter basis: FeCl_2_ × 4H_2_O 2.0g, EDTA 0.5 g, CuCl_2_ 0.03 g, H_3_BO_3_, ZnCl_2_, MnCl_2_ × 4H_2_O, (NH_4_)Mo_7_O_24_, AlCl_3_, CoCl_2_ × 6H_2_O, NiCl_2_, and 0.05 g, Na_2_S × 9H_2_O 0.3 g, and 1 mL of concentrated HCl. The medium was prepared by adding the buffer to distilled water containing resazurin, boiling for 10 min, and cooling under nitrogen flushing. The mixture was then transferred to serum bottles using the Hungate technique [71] and autoclaved (121 °C) for 60 min. All other components of the medium were added separately through filter (0.45 µm) sterilized solutions. All experiments were conducted at 65 °C and at pH 7.0 with a liquid–gas (L-G) ratio of 1:1 unless otherwise noted. In all cases, experiments were performed in triplicate.

### 3.3. Bacterial Strain

*Thermoanaerobacter* strain AK15 was isolated from an alkaline hot spring, Viti (pH 8.6; temperature 60 °C), in NE Iceland, according to Scully and co-workers [18]. The strain has been sequenced for 16S rRNA and is most closely related to *Thermoanaerobacter uzonensis* [18]. The strain was persevered in BM medium rigorously degassed by sonication under vacuum supplemented with 30% *v*/*v* glycerol and stored at −20 °C. All cultivations were conducted at pH 7.0 at 65 °C. All inoculation stocks of the strain were taken from frozen (−20 °C) culture with rigorously degassed 30% (*v*/*v*) glycerol and reactivated on BM medium containing glucose (20 mM). Reactivated cultures were inoculated (2% *v*/*v*) from exponential growth phase to 25 mL serum bottles (liquid-gas ratio 1:1). Cultures were grown for five days and screened for end-product formation.

### 3.4. Substrate Utilization Spectrum

The ability of strains to utilize selected hexoses (D-glucose, D-mannose, D-galactose), pentoses (D-xylose, D-arabinose, D-ribose), methypentoses (L-fucose and L-rhamnose), sugar alcohols (mannitol), and cellobiose were evaluated at a concentration of 20 mM, except for cellobiose (10 mM). Amino acids were tested at 20 mM concentration in the absence and presence of thiosulfate (concentration). Polymeric substrates (starch, cellulose, laminarin, xylan, chitosan, chitin, casein, keratin, collagen, lichenan, pectin, and keratin) were evaluated at a concentration of 0.2% (*w*/*v*), except laminarin (0.1% *w*/*v*). Cultures were incubated for a period of 5 days at which time end-products were analyzed. Experiments were conducted in 25 mL serum bottles with liquid–gas phase of 1.0 ratio. Control bottles were only with yeast extract (2 g L^−1^).

Samples of cellulose and complex biomass were obtained from Whatman paper, printed newspaper, grass (*Phleum pratense*), rhubarb (*Rheum rhabarbarum*), *Ascophyllum nodosum, Palmaria palmata, Laminaria digitata,* and *Ulva lactuca*. The biomass was pretreated as previously described with 0.1% H_2_SO_4_ and enzymes (CelluclastR and Novozyme 188) as previously described [31], rendering hydrolysates that were diluted to a concentration of 2.5% (*w*/*v*).

### 3.5. Influence of Liquid–Gas Phase Ratio

The influence of partial hydrogen pressure (*ρ*H_2_) on end-product formation was investigated with different ratios of liquid and gas phases when grown on 20 mM glucose. The liquid phase varied from 5.0, 29.45, and 50 mL in serum bottles with a total volume of 57 mL; thus, the L-G volume ratios were 0.09, 1.00, and 5.62, respectively. A control experiment at each L-G consisted of BM medium containing yeast extract (0.2% *w*/*v*) only. After 5 days of incubation, end-products were quantified by GC.

### 3.6. Analytical Methods

Hydrogen was analyzed by Perkin Elmer (Waltham, MA, USA) Auto System XL gas chromatograph, according to Orlygsson and Baldursson [70]. Alcohols and volatile fatty acids were measured by gas chromatography using a Perkin-Elmer (Waltham, MA, USA) Clarus 580 gas chromatograph, as previously described [70]. Lactate was quantified colorimetrically according to the method of Taylor [72] with modification according to Scully and Orlygsson [66]. Optical density was determined by measuring absorbance at 600 nm by a Shimadzu UV-1800 UV-Visible spectrophotometer in a quartz cuvette with a pathlength of 1 cm against a water blank. Hydrogen sulfide was analyzed as described by Cline [73].

## 4. Conclusions

The present study focuses on the fermentation of various substrates, both single carbohydrates and pretreated lignocellulosic biomass in batch culture with the main emphasis on producing bioethanol by a thermophilic anaerobic bacterium, *Thermoanaerobacter* strain AK15. More detailed investigations regarding the substrate spectrum covering amino acids, proteins, and macroalgae-associated biomolecules were also performed with the main aim of investigating the production of ethanol and other end-products from each substrate. The strain was found to be highly ethanologenic from most of the carbohydrates tested, including the constituents of lignocellulosic biomass, mannitol, and macroalgae biomass. It also produced long-chain alcohols from specific amino acids and could use volatile fatty acids as electron acceptors to produce their corresponding alcohols during carbohydrate degradation. The main limitations of using such a strain for biofuel and fine chemical production may lie in the normally low substrate concentrations that need to be used for thermophilic anaerobic bacteria, but this may be overcome through the optimization of culture conditions and by using fed-batch or continuous culture in future upscaling experiments. While this study takes a comprehensive examination of *Thermoanaerobacter* strain AK15–s ability to utilize a wide range of carbohydrates and amino acids, the main novelty of the study obtained lies, however, in the production of valuable alcohols from amino acids as well as the ability to convert volatile fatty acids to high-carbon fuel molecules using readily available and low-cost biomass.

## Figures and Tables

**Table 1:** Comparison of *Thermoanaerobacter* strain AK15 with selected species within the genus of *Thermoanaerobacter*.

	*T. ethanolicus*	*T. mathranii* subsp. *mathranii*	*T. pentosaceus*	*T. pseudoethanolicus*	*T. thermohydrosulfuricus*	*T. uzonensis*	*Thermoanaerobacter* Strain AK15
**Monosaccharides and polyols**							
Glucose	+	+	+	+	+	+	+
Galactose	+	−	+	NR	+	+	+
Mannose	+	+	+	NR	+	+	+
Fructose	+	+	+	NR	+	+	+
Arabinose	NR	+	+	NR	+	−	+
Xylose	+	+	+	+	+	+	+
L-fucose	−	NR	NR	NR	NR	NR	−
L-Rhamnose	NR	NR	NR	NR	NR	NR	−
Mannitol	−	+	+	NR	Var	+	+
**Di- and tri-saccharides**							
Cellobiose	+	+	+	+	+	+	+
Maltose	+	+	+	NR	+	+	+
Lactose	+	NR	NR	NR	+	−	+
Sucrose	+	+	+	+	+	+	+
Trehalose	−	+	NR	NR	+	+	NR
**Polysaccharides**							
Cellulose (filter paper)	−	−	+	−	−	−	−
Pectin	NR	−	NR	NR	+	−	−
Starch	+	+	+	+	+	−	+
Laminarin	NR	NR	NR	NR	NR	NR	−
Lichenin	NR	NR	NR	NR	NR	NR	−
Xylan	NR	+	+	NR	NR	+	−
Galactan	NR	NR	NR	NR	NR	NR	−
Mannan	NR	NR	NR	NR	NR	NR	−
Rhamnan	NR	NR	NR	NR	NR	NR	−
Reference	[7]	[22]	[30]	[27,31]	[23]	[32]	This study

+ = positive, − = negative, Var—variable, NR—not reported.

## Data Availability

Data are available in the Supplementary Materials associated with this work.

## Supplementary Materials

The following supporting information can be downloaded at: https://www.mdpi.com/article/10.3390/ijms25063490/s1.
