# Supplementary material for: Biotechnological Prospects of Thermoanerobacter AK15: End-Product Formation from Carbohydrates, Amino Acids, and Lignocellulosic and Macroalgae Hydrolysates
Source: Int J Mol Sci. 2024 Mar 20;25(6):3490. doi: 10.3390/ijms25063490 (PMC10971038; doi:10.3390/ijms25063490)
Supplement: Supplementary file 1 [file ijms-25-03490-s001.zip › ijms-2885575-supplementary.pdf]

Supplementary Tables.

Supplementary Table S1. Final optical density and pH from various substrates by *Thermoanaerobacter* strain AK15 after 5 days of cultivation. Values represent the average of triplicate measures ± standard deviation.

Carbon source	Optical density	pH
Control (yeast extract)	0.12±0.02	7.11±0.02
Cellobiose	0.42±0.02	6.21±0.04
Glucose	0.43±0.04	6.12±0.02
Galactose	0.48±0.03	6.21±0.03
Mannose	0.40±0.04	6.15±0.05
Xylose	0.35±0.06	6.12±0.04
Arabinose	0.42±0.16	6.23±0.03
Mannitol	0.45±0.03	6.21±0.04
Fucose	0.11±0.08	7.01±0.02
Rhamnose	0.10±0.04	7.06±0.06

Supplementary Table S2. Final optical density and pH from glucose degradation at different liquid-gas phase ratios by *Thermoanaerobacter* strain AK15 after 5 days of cultivation. Values represent the average of triplicate measures ± standard deviation.

Carbon source	Liquid-gas phase ratio	Optical density	pH
Control (yeast extract)	0.09	0.12±0.02	7.11±0.02
Control (yeast extract	1.00	0.12±0.03	7.21±0.01
Control (yeast extract	5.62	0.12±0.02	6.71±0.03
Glucose	0.09	0.32±0.05	6.05±0.02
Glucose	1.00	0.28±0.01	6.22±0.12
Glucose	5.62	0.31±0.01	6.45±0.16

Supplementary Table S3. Final optical density and pH of various polymeric substrates by *Thermoanaerobacter* strain AK15 after 5 days of cultivation. Values represent the average of triplicate measures ± standard deviation.

Carbon source	Optical density	pH
Control (yeast extract)	0.12±0.04	7.11±0.02
Starch	0.32±0.03	6.54±0.03
Cellulose	0.13±0.04	7.12±0.03
Laminarin	0.14±0.03	7.11±0.04
Xylan	0.14±0.04	7.15±0.02
Chitosan	0.15±0.01	7.12±0.05
Chitin	0.12±0.02	7.03±0.00
Lichenan	0.15±0.02	7.11±0.01
Pectin	0.11±0.03	7.01±0.02
Keratin	0.10±0.01	7.06±0.07
Mannan	0.18±0.02	6.90±0.05
Galactan	0.15±0.02	6.94±0.08
Rhamnan	0.17±0.01	6.92±0.05

Supplementary Table S4. Final optical density and pH of various hydrolysates from polymeric substrates by *Thermoanaerobacter* strain AK15 after 5 days of cultivation. Values represent the average of triplicate measures ± standard deviation.

Carbon source	Optical density	pH
Control (yeast extract)	0.12±0.04	7.11±0.02
Whatman paper	0.29±0.02	6.32±0.03
Newspaper	0.25±0.03	6.22±0.04
Timothy grass	0.33±0.01	6.31±0.01
Rhubarb leaves	0.19±0.02	6.23±0.01
Ascophyllum	0.34±0.02	6.27±0.02
Palmaria	0.21±0.02	6.23±0.02
Laminaria	0.43±0.01	6.26±0.04
Ulva	0.22±0.02	6.32±0.03

Supplementary Table S5. Final optical density and pH of the degradation of the branched-chain fatty acids in the absence and presence of thiosulfate by *Thermoanaerobacter* strain AK15 after 5 days of cultivation. Values represent the average of triplicate measures ± standard deviation.

Carbon source	Optical density	pH
Control	0.12±0.04	7.13±0.02
Control + S2O3	0.23±0.03	6.87±0.04
Leucine	0.13±0.02	7.03±0.02
Leucine + S2O3	1.31±0.29	6.23±0.06
Isoleucine	0.14±0.04	7.05±0.02
Isoleucine + S2O3	1.47±0.13	6.04±0.06
Valine	0.13±0.03	7.02±0.03
Valine + S2O3	1.51±0.17	6.12±0.06
